# Octadecadienoate derivatives from *Michelia champaca* seed extract as potential larvicide and pupicide against Dengue vector *Aedes albopictus*

**DOI:** 10.1186/s13104-023-06487-9

**Published:** 2023-09-12

**Authors:** Manali Dutta, Goutam Chandra

**Affiliations:** Mosquito, Microbiology and Nanotechnology Research Units, Parasitology Laboratory, Department of Zoology, The University of Burdwan, Burdwan, West Bengal 713104 India

**Keywords:** *Michelia champaca*, *Aedes albopictus*, Larvicide, Pupicide, Bioactive compound, Non-target organisms

## Abstract

The present study was designed aiming at finding novel botanicals for controlling the vector population. Objective was to evaluate the larvicidal and pupicidal efficacies of crude and solvent extracts of *Michelia champaca* seed against the notorious dengue vector *Aedes albopictus*. 0.5% concentration of the crude extractive and 40 ppm concentration of ethyl acetate extractive were enough to execute 100% of larval mortality of all the instars after 72 h of exposure and the LC_50_ and LC_90_values (95% confidence level) of ethyl acetate extractive were 0.9880 ppm and 36.0491 ppm. In case of pupicidal bioassay, 100% mortality was observed at 200 ppm of ethyl acetate extract. Through TLC techniques, the bioactive compounds were isolated, which caused remarkable larval toxicity at 15 ppm concentration. Three-way factorial ANOVA analysis showed different concentrations, time intervals, and instars revealed a significant difference in larval death. FT-IR analysis revealed the presence several important functional groups. Presence of methyl 5,12-octadecadienoate and ethyl 9cis,11trans-octadecadienoate were ascertained by GC-MS analysis. The said bioactive compounds showed very low toxicity in non-target organisms such as damselfly (*Ischnura* sp.) and water bug (*Diplonychus* sp.) Thus, proclaiming the potentialities of *Michelia champaca* seed extracts as larvicidal and pupicidal agents against *Ae. albopictus.*

## Introduction

The dengue transmitter *Aedes albopictus* is specifically distributed in the tropical and sub-tropical zones of the world. Adult females of *Ae. albopictus* being anthropophilic, targets human as hosts for their blood meal. By feeding on infected humans, *Aedes* mosquitoes get infected by the dengue virus, which they carry with them for the rest of their lives and due to transovarial transmission of the dengue virus it remains in the mosquito population [1]. Control of mosquito has become a serious concern because it transmits life threatening diseases like Dengue Hemorrhagic Fever (DHF), Chikungunya, yellow fever, and Zika [2]. Moreover, dengue hemorrhagic fever enlists itself among the 10 most leading factor of hospitalization in more than eight Asian countries [3]. Most of the control strategies are directed towards the use of chemical mosquitocides such as organochlorines, organophosphates and pyrethroids because of their rapid effectiveness, but they are not only nonselective but also cause hazards to various non target organisms [4]. Therefore, their continuous utilization may lead to environmental imbalance and to develop resistance among mosquitoes [5–7]. Thus, in order to address such discrepancies new efforts have been made to control mosquito biologically [8–10] or by using plant-based phytochemicals to formulate larvicides [11], pupicides [12], adulticides as well as repellents [13, 14].

*Michelia champaca* L. originating from South and Southeast Asia, is a large evergreen tree belonging to the family: Magnoliaceae, that is predominantly grown for its timber, specifically known for its aromatic blossoms which flowers during the monsoon. Birds are particularly attracted to the aril-covered seeds of this tree [15]. From the traditional literature it has been known to be used as a febrifuge and for treatment of fever, leprosy, postpartum protection, and delivery. It is also known to have antipyretic and anti-inflammatory properties [16]. Moreover, the family of Magnoliaceae comprises of various aromatic plant species that is reported to have anti-pest and mosquitocidal activities. Referring to the study conducted by Luu-Dam et al., it had been highlighted that *Magnolia citrata* Noot and Chalermglin (Magnoliaceae) essential oil do possess the insecticidal and attractant properties against two important pests: the yellow fever mosquito *Aedes aegypti* and the Mediterranean fruit fly *Ceratitis capitata* [17]. The larvicidal properties of hexane and methanolic fruit extracts from *Magnolia salicifolia* were confirmed by Kelm et al. against *Ae. aegypti* [18]. Likewise, the insecticidal activity of essential oils from various parts of *Magnolia grandiflora* demonstrated the most potent toxic effects against *Ae. aegypti* larvae [19]. Wang et al. documented the larvicidal property of the substances derived from the hydro-distillation of seeds belonging to the species *Magnolia denudate* (native to China) against 4 mosquito species *Culex pipens pallens, Ae. aegypti, Ae. albopictus*, and *Anopheles sinensis* [20]. There are also several other examples regarding the anti pest properties of different species of *Magnoliaceae* spp [21].

Thus, the present study aimed at evaluating the larvicidal and pupicidal efficacies of the seed extracts of *M. champaca* against all the larval and pupal stages of *Ae. albopictus* mosquito which proved to be a novel source of mosquitocide till date.

## Materials and methods

### Collection of seeds

Fresh and green fruits of *M. champaca* were collected randomly during July-October, 2020 from the plants growing along the Chandan Nagar strand road, Hooghly, West Bengal, India. The plant along with seeds was accurately identified by Dr. Ambarish Mukherjee and the specimen copy with Voucher No. GCMD/2018/S002 was submitted at the departmental herbarium (BURD), Department of BOTANY, The University of Burdwan. The collected fruits were cleansed in distilled water, slit opened to obtain the seeds discarding the pericarps. These seeds were further processed for crude extract and solvent extract preparation. Moreover, when the fruits get ripened, they rupture and the seeds get dispersed thereby increases the difficulty of seed collection. So, the intact mature fruits were collected for the purpose.

### Collection and rearing of larvae

The present experimentation was conducted at the University of Burdwan, West Bengal, India (23° 16’ N,87° 54’E). *Ae. albopictus* larvae growing in nearby breeding sites such as open tanks, earthen pots, ice cream cups, old shoes etc. were collected and cultured in specific plastic trays filled with tap water. A combination of brewer’s yeast, dog biscuit and pond algae were feed to the larvae. The reared larvae were protected from any pathogens, insecticides or repellents. Adult mosquitos upon emergence were transferred to cages and regularly provided with 10% glucose solution. The whole experimental setup was maintained at 25 ± 2 °C at 80–85% relative humidity under a 14:10 light and dark photo period.

### Crude extract preparation

The seeds were collected from the mature fruits of *M. champaca* and properly rinsed in distilled water. Washed seeds were soaked in bloating paper to make water free and crushed within an electric grinder to prepare a smooth paste. The paste was squeezed to obtain the sap which was filtered through Whatmann No.1 filter paper. The filtrate was treated as the stock solution to prepare graded crude extract concentration (0.1%,0.2%,0.3%,0.4% and 0.5%) by adding distilled water.

### Solvent extract preparation

The properly washed seeds were chopped and shade dried at room temperature for 20 days. 200 gm of dried chopped seeds were transferred into the thimble of Soxhlet apparatus. 2 L of each of the three different solvents (ethyl acetate, chloroform:methanol and methanol) were used sequentially to prepare three different extractives. Each time the elutes were collected, filtered and stored in glass beakers. The extracts were properly dried using rotary evaporator and preserved at 4 °C temperature.

### Dose dependent larvicidal bioassay

Larvicidal bioassay was carried out using both the crude and the solvent extracts following the standard protocol of World Health Organization. For the bioassay 100 ml of distilled water was added to each 100 ml sterilized beaker and graded concentration of crude extract of the seeds of *M. champaca* ranging from 0.1 to 0.5% were added into them with meticulous care. 25 larvae of all the four instars (1st, 2nd, 3rd, 4th) of *Ae. albopictus* were separately transferred to the beakers having graded concentrations. Likewise solvent extractives were used to prepare five graded concentrations ranging from 10 ppm to 50 ppm and administered against 3rd instar larvae and the most efficacious solvent was ascertained from this. After this the most effective extractive was charged against all the larval instars in the aforesaid graded concentration pattern. Larval mortality was noted after 24, 48, 72 h of exposure. Larvae were confirmed to be dead when they remained unresponsive upon pricking with needle in the siphon and cervical zone. Each experiment was triplicated on 3 different days (n = 9) with three control sets under laboratory conditions.

### Dose dependent pupicidal bioassay

Graded concentrations of the most efficacious solvent extract (50ppm, 100ppm, 150ppm, 200ppm, 250ppm) were prepared and added into separate sterilized beakers containing 100ml of distilled water. 25 early pupae of *Ae. albopictus* were transferred to each experimental beaker. Pupal mortality rate was recorded after 24, 48 and 72 h of exposure. The study was replicated thrice with three control sets.

### Semi-field experimentation

Following the standard protocol, the most efficacious solvent extract of the seeds of *M. champaca* were administered against *Ae. albopictus* larvae under semi-field condition [22, 23]. 5 glass jars with a capacity of 5 L were half-buried in the ground in a semi-field condition. Each container received 4990 mL of clear water and 10 ml of ethyl acetate seed extract of graded concentrations to make 100 ppm, 200ppm, 300 ppm, 400 ppm and 500 ppm container solutions. As a control, an extra glass jar containing 5 L of water were used. One hundred 3rd instar *Ae. albopictus* larvae were released in each glass jar, and larval mortality was recorded after 24, 48 and 72 h of exposure. The jars were covered using a mosquito net to ensure that no wild mosquitoes laid eggs in the jars. Three such set ups were arranged on each experimental day and the entire experimentation was performed thrice on three different days (each with 3 set ups).

### Phytochemicals analyses

Following the standard protocols led by Harbone and Sofowara [24, 25] various photochemicals analyses in the crude as well as ethyl acetate extractive of *M. champaca* seeds were performed to ascertain the presence of sterols, flavonoids, saponins, terpenoids, glycosides and tannins.

### Thin layer chromatography (TLC)

The most effective ethyl acetate extractive of *M. champaca* seeds was monitored by utilizing thin-layer chromatographic techniques where 0.5 mm thick glass plates were coated with silica gel. Silica gel - G weighing 50 g was placed in a 500 ml glass beaker equipped with a glass lid. To this, 100 ml of distilled water was added. The mixture was vigorously shaken and transferred into a spreader. Depending on the requirements, the spreader was adjusted to 0.5 mm scale, and quickly drawn over the glass plates. These silica gel-coated plates were initially left in the air and subsequently placed in a hot-air oven at a temperature of 110 °C for 30 min to activate them. A total of 25 glass plates were prepared in this manner before conducting thin-layer chromatography (TLC). The bioactive ingredients present in the extractive was separated using mobile phase of petroleum ether, n-hexane and ethyl acetate in a specific ratio, placed inside the solvent chamber. After 50 min, the plates were removed from the solvent chamber, and the solvent was allowed to evaporate in a dry place. The glass plates were then placed in an iodine chamber (21 × 21 × 9 cm) for one minute. After that, the plate was withdrawn from the chamber, and the primary bands that occurred on similar R_f_ values were combined and employed as an apparently pure substance. The distance of run of the developing solution from the plate bottom, as well as the distance between the sample spot and the bottom of the plate was measured. The R_f_, value was determined using the following formula:

$$ {\text{R}}_{\text{f}}= \frac{\text{d}\text{i}\text{s}\text{t}\text{a}\text{n}\text{c}\text{e}\, \text{o}\text{f}\, \text{t}\text{h}\text{e}\, \text{s}\text{p}\text{o}\text{t}\, \text{c}\text{e}\text{n}\text{t}\text{r}\text{e}\, \text{f}\text{r}\text{o}\text{m}\, \text{t}\text{h}\text{e}\, \text{s}\text{t}\text{a}\text{r}\text{t}\, \text{p}\text{o}\text{i}\text{n}\text{t}}{ \text{d}\text{i}\text{s}\text{t}\text{a}\text{n}\text{c}\text{e}\, \text{o}\text{f}\, \text{t}\text{h}\text{e}\, \text{s}\text{o}\text{l}\text{v}\text{e}\text{n}\text{t}\, \text{r}\text{u}\text{n}\, \text{f}\text{r}\text{o}\text{m}\, \text{t}\text{h}\text{e}\, \text{s}\text{t}\text{a}\text{r}\text{t}\, \text{p}\text{o}\text{i}\text{n}\text{t}}$$

Each spot with similar R_f_ value was collected from 25 chromatogrammed plates and dissolved in 25 cc of absolute alcohol, before being heated in a water bath (temp: 60–65 °C) for 5 min. The precipitate comprising silica gel was discarded and a transparent solution was taken in a separate conical flask. After evaporation of the alcohol, the solid mass that had accumulated at the bottom of the conical flask was scraped out and preserved for further analytical studies.

### Fourier transform InfraRed (FT-IR) Spectroscopy, and Gas Chromatography- Mass Spectroscopy (GC-MS) analyses for determination of active ingredient

To analyze the dried sample containing the active ingredient, a portion of it was examined using Fourier transform infrared (FT-IR) spectroscopy. The sample was placed in a vacuum desiccator with potassium hydroxide (KOH) pellets for 48 h to ensure optimal conditions. Subsequently, FT-IR spectral analyses were conducted using a FT-IR Spectrometer (JASCO FT/IR: Model 4700) with potassium bromide (KBr) pellets as blank reference. This was used to identify the important functional groups present in the bioactive compound extracted from the spot.

The purified fraction was subjected to GC-MS analysis (Perkin Elmer, Model: Clarus 680 GC & amp; Clarus 600 C MS comprising a liquid autosampler) for additional biochemical characterization. The analysis was performed under specific conditions: a capillary column (Elite- 5MS) measuring 60 m in length, 0.25 mm in diameter, and 0.25 μm in film thickness was used in electronic mode with an electron energy of 70 eV. Helium (99.999%) was employed as the carrier gas, flowing at a constant rate of 1 ml/min, with an injection volume of 1.0 µl. The injector temperature was set at 280 °C, while the ion source temperature was maintained at 180 °C. The oven temperature was programmed at 60 °C with an increase at the rate of 7 °C per minute. Mass spectra were recorded at 70 eV with a total run time of 39 min. The peaks so obtained was analyzed by data analysis software NIST-2014.

### Bioassay using active ingredient

The larvicidal bio test was performed on early 3rd instar larvae using a standard method [26]. Four distinct bioactive chemical test concentrations (5ppm, 10 ppm, 15 ppm, and 20 ppm) were used. On three distinct days (n = 9), each experiment was repeated three times with three sets of controls.

### Evaluation of impact on non-target populations

Odonatan and hemipteran nymphs such as damselfly (*Ischnura* sp.) and water bug (*Diplonychus* sp.) were chosen as nontarget organisms since they usually share the same habitats with mosquito immatures. To acclimate these organisms to the laboratory environment, they were placed in surroundings similar to their natural habitats. Following the procedure outlined by Suwannee et al. [27], the non-target organisms were exposed to the bioactive compound at the LC_50_ concentration (determined for early 3rd instar larvae after 24 h). In the experiment, 25 of the 3rd instar *Ischnura* sp. naiads were placed in a 500 ml beaker containing 200 ml of pond water, while 25 early 3rd instar nymphs of *Diplonychus* sp. were placed in a 12.6 × 10 × 6-inch plastic tray filled with pond water. The number of deceased non-target organisms was recorded after 24, 48, and 72 h of exposure, and the mortality rate was calculated as a percentage. The experiments were conducted over three separate days, with three replicates performed each day for each organism (n = 9). Additionally, a control group (not exposed to the test solution) was included in parallel with the experiment each day (n = 3), and the average mortality rates were documented.

### Statistical analyses

Abbott’s Formula was used to correct the percentage mortalities (% M) [28]. “MS Excel 2010” was also used for regression analysis, while “Stat plus 2009 professional” was used for log probit [29] and ANOVA studies.

## Results

### Larvicidal bioassay

After 72 h of exposure, crude seed extract showed 100% mortality against 1st, 2nd and 4th instar larvae of *Ae. albopictus* at 0.4%concentration (Table 1), while 0.5% crude extract was enough to execute 100% mortality in 3rd instar larval population. Ethyl acetate extractive of *M. champaca* showed the strongest larvicidal function against 2nd instar *Ae. albopictus* larvae (Table 2). Ethyl acetate extractive demonstrated 100% mortality against all the larval instars at 40 ppm concentration after 72 h of exposure (Table 2). Death rate of each larval instar increased as the exposure time increased. A reversely proportional relationship between time of exposure and LC values was shown by log probit analysis (95% confidence level). After 72 h of exposure, the lowest LC_50_, and LC_90_, levels were reported in 1st instar larvae. The lowest recorded value of LC_50_ and LC_90_ on crude extract were 0.0337% and 0.3708% respectively (Table 3). The lowest LC_50_ and LC_90_ values of ethyl acetate extractive were 0.9880 ppm and 36.0491 ppm respectively (Table 4). According to both of the regression analyses, the mortality rate (Y) had a positive connection with the concentration (x) of extracts with a regression coefficient (R^2^) near to 1 in most cases, (Tables 3 and 4). The three-way factorial ANOVA of ethyl acetate extractive (Table 5) of *M. champaca* seed examined with different concentrations, time intervals, and instars revealed a significant difference in larval death (p < 0.05) with relation to these three variables.

**Table 1:** Larvicidal bioassay using the crude seed extractive of *Michelia champaca* against all the four instars of *Aedes albopictus*

Larval Instars	Concentration (%)	Percent Mortality (Mean ± SE)		
		24 h	48 h	72 h
First	0.1	25.33 ± 0.33	34.67 ± 0.33	54.67 ± 0.33
	0.2	33.33 ± 0.33	53.33 ± 0.66	72.00 ± 0.58
	0.3	53.33 ± 0.33	72.00 ± 0.58	81.33 ± 0.33
	0.4	66.66 ± 0.33	98.67 ± 0.33	**100.00 ± 0.00**
	0.5	81.33 ± 0.33	**100.00 ± 0.00**	**100.00 ± 0.00**
Second	0.1	24.00 ± 0.00	36.00 ± 0.00	42.67 ± 0.33
	0.2	34.67 ± 0.33	57.33 ± 0.67	72.00 ± 0.58
	0.3	54.67 ± 0.33	70.67 ± 0.33	81.33 ± 0.33
	0.4	70.67 ± 0.33	94.67 ± 0.33	**100.00 ± 0.00**
	0.5	84.00 ± 0.00	**100.00 ± 0.00**	**100.00 ± 0.00**
Third	0.1	20.00 ± 0.00	32.00 ± 0.00	42.67 ± 0.33
	0.2	33.33 ± 0.33	56.00 ± 0.58	70.67 ± 0.33
	0.3	52.00 ± 0.00	72.00 ± 0.00	81.33 ± 0.33
	0.4	65.33 ± 0.33	89.33 ± 0.33	98.67 ± 0.33
	0.5	82.67 ± 0.33	98.67 ± 0.33	**100.00 ± 0.00**
Fourth	0.1	21.33 ± 0.33	29.33 ± 0.33	40.00 ± 0.00
	0.2	36.00 ± 0.58	53.33 ± 0.33	84.00 ± 0.58
	0.3	52.00 ± 0.58	69.33 ± 0.33	94.67 ± 0.33
	0.4	69.33 ± 0.33	92.00 ± 0.00	**100.00 ± 0.00**
	0.5	77.33 ± 0.33	**100.00 ± 0.00**	**100.00 ± 0.00**

The highest mortality, essential LC_50_ values are made in bold

**Table 2:** Larval mortality of all the four instars of *Aedes albopictus* caused due to their exposure to ethyl acetate extractive of *Michelia champaca*

Larval Instars	Concentration (ppm)	Percent Mortality (Mean ± SE)		
		24 h	48 h	72 h
First	10	25.33 ± 0.33	42.67 ± 0.33	58.67 ± 0.33
	20	33.33 ± 0.33	62.67 ± 0.33	74.67 ± 0.33
	30	53.33 ± 0.33	74.67 ± 0.33	84.00 ± 0.58
	40	66.67 ± 0.33	98.67 ± 0.33	**100.00 ± 0.00**
	50	81.33 ± 0.33	**100.00 ± 0.00**	**100.00 ± 0.00**
Second	10	24.00 ± 0.00	37.33 ± 0.33	45.33 ± 0.33
	20	34.67 ± 0.33	58.67 ± 0.33	76.00 ± 0.00
	30	54.67 ± 0.33	86.67 ± 0.33	98.67 ± 0.33
	40	70.67 ± 0.33	**100.00 ± 0.00**	**100.00 ± 0.00**
	50	84.00 ± 0.33	**100.00 ± 0.00**	**100.00 ± 0.00**
Third	10	20.00 ± 0.33	32.00 ± 0.00	48.00 ± 0.58
	20	33.33 ± 0.33	56.00 ± 0.58	73.33 ± 0.33
	30	52.00 ± 0.33	73.33 ± 0.33	89.33 ± 0.33
	40	65.33 ± 0.58	88.00 ± 0.58	**100.00 ± 0.00**
	50	82.67 ± 0.33	**100.00 ± 0.00**	**100.00 ± 0.00**
Fourth	10	21.33 ± 0.33	34.67 ± 0.33	62.67 ± 0.33
	20	36.00 ± 0.00	54.67 ± 0.33	77.33 ± 0.33
	30	52.00 ± 0.00	70.67 ± 0.33	92.00 ± 0.58
	40	69.33 ± 0.33	90.67 ± 0.33	**100.00 ± 0.00**
	50	77.33 ± 0.00	96.00 ± 0.58	**100.00 ± 0.00**

The highest mortality, essential LC_50_ values are made in bold

**Table 3:** Regression and log-probit analyses using the crude seed extractive of *M. champaca* against *Ae. albopictus*

Larval Instars	Period of Exposure	LC _50_ (%)	LC _90_ (%)	Regression	R^2^-value
1st	24	0.2862	0.5614	Y = 145.33x + 8.4	0.9884
	48	0.1765	0.4039	Y = 176x + 18.933	0.9569
	72	0.0337	0.3708	Y = 118.67x + 46	0.9421
2nd	24	0.2769	0.5333	Y = 156x + 6.8	0.9928
	48	0.1685	0.4105	Y = 165.33x + 22.133	0.9727
	72	0.0953	0.3757	Y = 142.67x + 36.4	0.9021
3rd	24	0.2956	0.5498	Y = 157.33x + 3.5	0.9974
	48	0.1824	0.4224	Y = 166.67x + 19.60	0.9785
	72	0.0990	0.3794	Y = 142.67x + 35.87	0.9159
4th	24	0.2917	0.5670	Y = 145.33x + 7.6	0.9891
	48	0.1955	0.4178	Y = 180x + 14.8	0.9792
	72	0.0520	0.3461	Y = 136x + 42.933	0.7221

**Table 4:** Regression and log-probit analyses using the ethyl acetate extractive of *M. champaca* seeds against *Ae. albopictus*

Larval Instars	Period of Exposure	LC _50_ (ppm)	LC _90_ (ppm)	Regression	R^2^-value
1st	24	27.1363	56.1481	Y = 1.4533x + 8.4	0.9884
	48	12.9203	39.4684	Y = 1.5067x + 30.533	0.9538
	72	**0.9880**	**36.0491**	**Y = 1.08x + 51.067**	**0.9410**
2nd	24	27.6923	53.3333	Y = 1.56x + 6.8	0.9928
	48	14.0799	38.0794	Y = 1.6667x + 26.533	0.9078
	72	4.5001	34.5009	Y = 1.3333x + 44	0.7776
3rd	24	29.5767	55.0009	Y = 1.5733x + 3.467	0.9974
	48	18.1744	41.9839	Y = 1.68x + 19.467	0.9817
	**72**	**5.4083**	**36.0197**	**Y = 1.3067x + 42.933**	**0.8834**
4th	24	29.1750	56.6985	Y = 1.4533x + 7.6	0.9891
	48	17.8150	43.0245	Y = 1.5867x + 21.733	0.9739
	72	7.3975	33.6998	Y = 0.9733x + 57.2	0.9051

The highest mortality, essential LC_50_ values are made in bold

**Table 5:** Three-way ANOVA analysis of mortality of all the four larval instars, different hours of exposure and different concentrations of ethyl acetate extract as variables

Source of variation	Sum of squares (SS)	Degree of freedom (df)	Mean of squares (MS)	F value	p-level
Instars (I)	114.06	3	38.0208	17.0950	0.00
h (H)	1,468.97	2	734.4894	330.2427	0.00
Conc. (C)	4,448.78	4	1,112.1962	500.0681	0.00
I × H	85.48	6	14.2483	6.4063	0.00
I × C	47.96	12	3.9970	1.7972	0.056
H × C	165.43	8	20.6774	9.2970	0.00
I × H × C	82.78	24	3.4491	1.5508	0.064
Within groups	264.67	119	2.2241	---	---
Total	6,678.15	178	37.5177	-- --	---

### Pupal toxicity

Ethyl acetate extractive of *M. champaca* had excellent pupicidal efficacy. After 48 h of exposure, cent percent pupal mortality was reported at a concentration of 200 ppm, with LC_50_ and LC_90_ values of 62.38ppm and 120.87 ppm respectively (Table 6).

**Table 6:** Percent mortality, probit and regression analyses of *Ae. albopictus* pupae treated with ethyl acetate extract of *M. champaca* seeds

Concentration (ppm)	Time of exposure (hr)	Percent mortality (Mean ± SE)	LC _50_ (ppm)	LC _90_ (ppm)	Regression	R^2^-value
**50**	**24**	53.33 ± 0.33	81.11	215.25	Y = 0.2134x + 42.664	0.99
**100**		62.67 ± 0.54				
**150**		76.00 ± 0.33				
**200**		86.67 ± 1.20				
**250**		94.67 ± 0.33				
**50**	**48**	74.67 ± 0.88	**62.38**	**120.87**	0.1333x + 70.136	0.94
**100**		84.00 ± 0.67				
**150**		92.00 ± 0.00				
**200**		**100.00 ± 0.00**				
**250**		**100.00 ± 0.00**				

The highest mortality, essential LC_50_ values are made in bold

### Phytochemical analyses

The crude seed extract of *M. champaca* showed the presence of tannin, terpenoids, saponin, flavonoids, alkaloids, coumarins, cardiac glycosides and glycosides while the ethyl acetate extractive comprised all the above, except glycosides and cardiac glycosides (Table 7).

**Table 7:** Results of the phytochemical analyses using both crude and ethyl acetate extracts of *M. champaca*

Phytochemicals	Crude extract	Ethyl acetate extract
Tannin	**+**	**+**
Terpenoids	**+**	**+**
Sterols		
Saponin	**+**	**+**
Flavonoids	**+**	**+**
Alkaloids	**+**	**+**
Coumarins	**+**	**+**
Glycosides	**+**	
Cardiac glycosides	**+**	

### Isolated bioactive fraction and its larvicidal efficacy

**Fig. 1:**
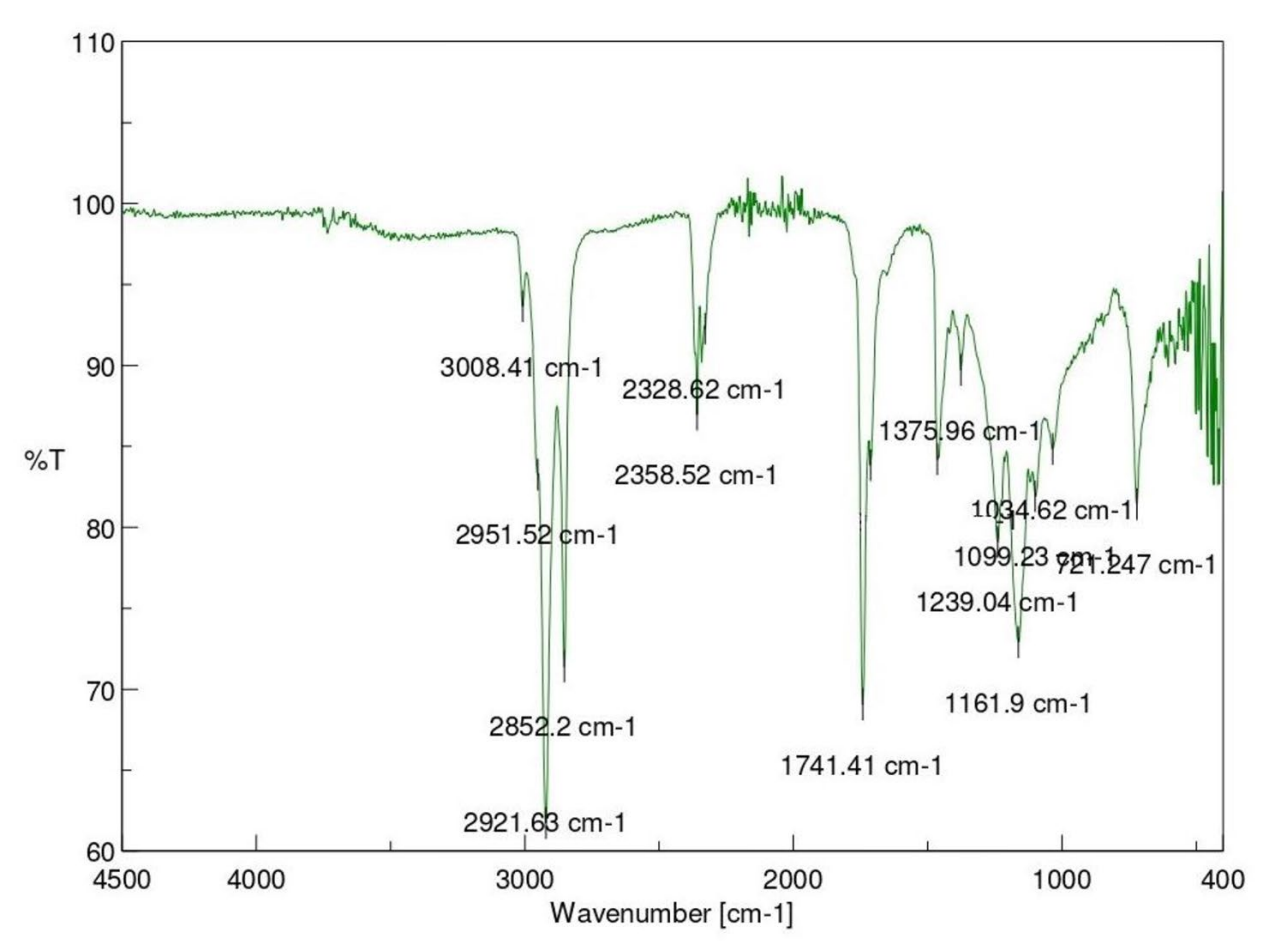
IR analysis of the active ingredient obtained from TLC fraction of the ethyl acetate extract of *Michelia champaca* seeds

**Fig. 2:**
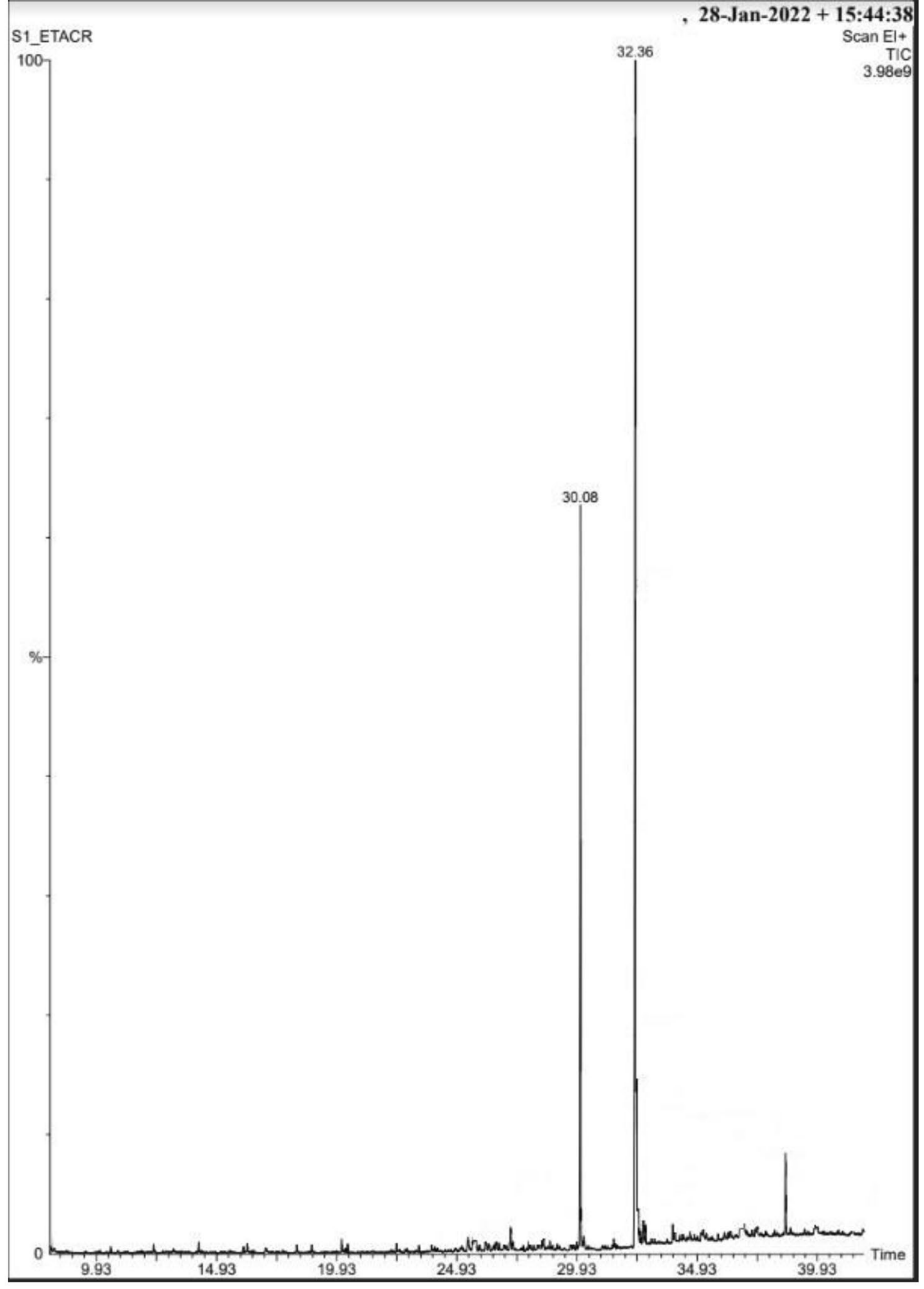
Graph obtained from GCMS analysis of the isolated bioactive compounds from ethyl acetate extract of *Michelia champaca* seeds

**Fig. 3:**
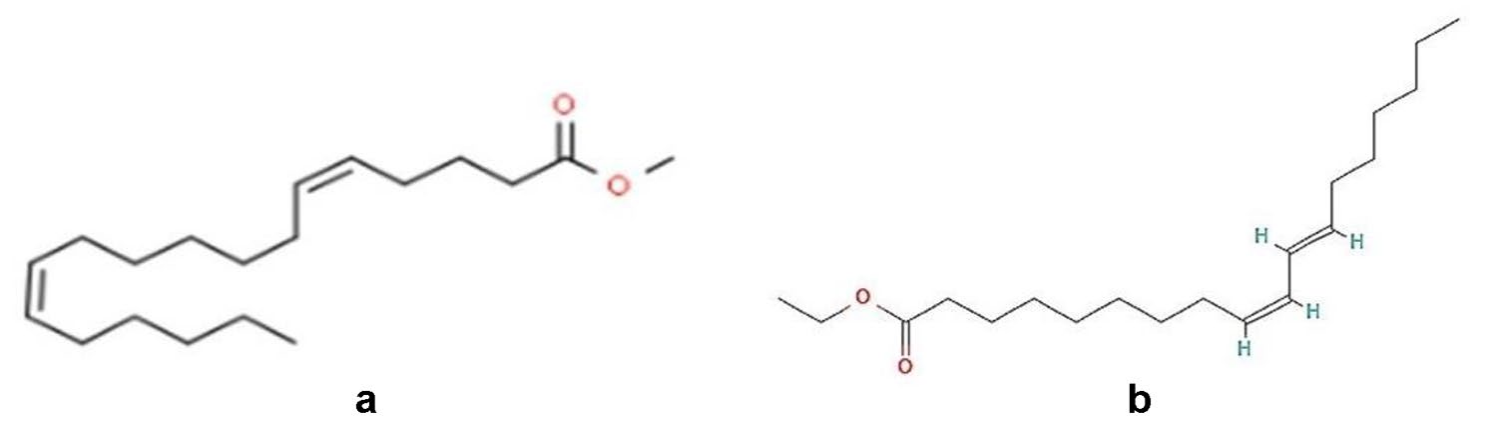
(**a**): methyl 5,12-octadecadienoate. (**b**): ethyl 9 cis, 11 trans-octadecadienoate

Infrared Spectroscopy analysis revealed the presence of functional groups- aldehydes, aromatics, amine, amides, ketone, esters and anhydrides in TLC fraction of *M. champaca* seeds (Fig: 1). The bioactive compound obtained from the GC-MS analysis (Fig: 2) was methyl 5,12-octadecadienoate and ethyl 9 cis,11trans-octadecadienoate having molecular weight- 294 and 308 respectively [Fig: 3(a) and 3(b)]. Table 8 presents the mortality rates of 3rd instar test mosquito larvae with the isolated compounds (R_f_= 0.46), which depicts cent percent mortality at 15 ppm concentration after 72 h of exposure.

**Table 8:** Mortality rate of third instar larvae of *Ae. albopictus* against ethyl acetate extract TLC fraction with R_f_ value 0.46 isolated from the seeds of *M. Champaca*

Concentration (ppm)	Percent Mortality (Mean ± SE)		
	24 h	48 h	72 h
5	45.57 ± 0.33	57.33 ± 0.67	70.67 ± 0.33
10	65.00 ± 0.57	73.33 ± 0.57	85.00 ± 0.57
15	83.24 ± 0.33	93.33 ± 0.33	**100.00 ± 0.00**
20	92.00 ± 0.00	**100.00 ± 0.00**	**100.00 ± 0.00**

### Effect on non-target organisms

No or minimal mortality or abnormality in behavior was accounted for tested non-target organisms. Isolated bioactive ingredients showed about 1.00% mortality in *Ischnura* naiads but no death was observed against *Diplonychus* sp. after 72 h of exposure (Table 9).

**Table 9:** Effect of Bio active compounds isolated from ethyl acetate extract of *M. Champaca* seeds on non-target organisms at laboratory conditions

Non-Target Organism (Nymphs)	Percent Mortality (Mean ± SE)		
	24 h	48 h	72 h
***Diplonychus*** **spp.**	0.00 ± 0.00	0.00 ± 0.00	0.00 ± 0.00
**Control**	0.00 ± 0.00	0.00 ± 0.00	0.00 ± 0.00
***Ischnura*** **spp.**	0.00 ± 0.00	1.00 ± 0.00	1.00 ± 0.00
**Control**	0.00 ± 0.00	0.00 ± 0.00	0.00 ± 0.00

### Semi-field experiment

As demonstrated in Table 10, at simulated semi-field circumstances, the ethyl acetate extractive of *M. champaca* seeds exhibited promising larvicidal property against 3rd instar *Ae. albopictus* larvae. At 500 ppm concentration of the said extractive 98.50% larval mortality had been achieved after 72 h of exposure. On probit analysis, the LC_50_ and LC_90_ values were recorded to be as low as 161.86 ppm and 390.77 ppm at semi-field conditions.

**Table 10:** Larvicidal efficacy of ethyl acetate extract of *M. champaca* seeds against *Ae. albopictus* larvae under simulated semi field condition

Time of exposure (Hour)	Concentration (ppm)	Day 1	Day 2	Day 3	Average mortality	LC_50_ (ppm)	LC_90_ (ppm)
		Percent mortality (Mean ± SE)					
**24 h**	**100**	26.00 ± 0.00	25.33 ± 0.33	26.67 ± 0.67	26.00	290.32	1043.72
	**200**	37.33 ± 0.88	34.50 ± 1.45	36.67 ± 0.58	36.17		
	**300**	52.50 ± 0.88	50.00 ± 0.67	50.67 ± 0.33	51.07		
	**400**	61.67 ± 0.88	59.57 ± 1.12	62.00 ± 0.33	61.08		
	**500**	72.33 ± 0.33	70.88 ± 0.67	72.00 ± 0.33	71.74		
	**Control**	1.33 ± 0.33	3.00 ± 0.67	1.33 ± 0.33	1.88		
**48 h**	**100**	39.67 ± 0.67	40.50 ± 1.20	42.00 ± 1.15	40.73	184.63	650.41
	**200**	53.33 ± 0.33	55.67 ± 1.15	56.67 ± 0.67	55.23		
	**300**	66.67 ± 0.67	65.50 ± 0.88	67.87 ± 0.33	66.68		
	**400**	74.00 ± 0.00	75.33 ± 1.20	77.67 ± 0.67	75.67		
	**500**	88.33 ± 0.33	87.67 ± 0.67	85.50 ± 1.12	87.17		
	**Control**	6.00 ± 0.00	08.00 ± 1.15	7.33 ± 0.33	7.11		
**72 h**	**100**	52.50 ± 0.88	54.00 ± 0.67	53.33 ± 0.33	53.27	**161.86**	**390.77**
	**200**	65.67 ± 0.33	66.67 ± 1.12	64.00 ± 0.88	65.45		
	**300**	78.50 ± 0.67	76.33 ± 1.15	78.00 ± 0.00	77.61		
	**400**	87.67 ± 1.12	89.00 ± 0.33	88.57 ± 1.12	88.41		
	**500**	97.33 ± 0.33	98.00 ± 0.67	**98.50 ± 0.88**	97.94		
	**Control**	09.67 ± 0.67	11.33 ± 0.33	10.67 ± 0.67	10.56		

The highest mortality, essential LC_50_ values are made in bold

## Discussion

Transovarial transmission of various disease-causing viruses found in *Aedes* mosquitoes makes them more threatening to humans and other lifeforms. Thus, claiming the need for vector control is of grave concern. The larval and the pupal instars are usually targeted due to their confinement in the water bodies. The plant kingdom contains a vast and unexplored reservoir of phytochemicals that could be effectively utilized instead of synthetic insecticides in mosquito control programs. A wide range of trees and shrubs have been discovered to possess phytochemicals that have the potential to control mosquito populations. Kishore et al. [30] authored a review on the effectiveness of phytochemicals against mosquito larvae based on their chemical properties. The environment friendly natural mosquito larvicidal botanicals have competed out the old use of toxic chemical insecticides in all spheres. Advantages that gained interest are being target specificity, low-toxic to other lifeforms, biodegradable and do not lead to bioaccumulation [31]. Plants served as traditional source of pesticides as they evolved various adaptive measures for better survival and reproduction [32]. Besides, botanicals have been reported to have antibacterial [33, 34] anthelmintic [35, 36] and many other useful activities.

Previous literature depicts various researchers have worked with plant extractives to control mosquito vectors, According to Rahuman and Venkatesan [37] methanol extractives of *Cannabis sativus*, *Cannabis indica*, *Momordica charantia*, petroleum ether extractive of *Citrullus colocynthis*, and acetone extractive of *Trichosanthes anguina* showed larvicidal efficacy against *Ae. aegypti* with LC_50_ values 492.73, 309.46, 199.14, 74.57 and 554.20 ppm respectively. Baranitharan and Dhanasekaran [38] showed that 50% lethality of *Ae. aegypti* was caused at 97.19 mg/dl conc. of ethyl acetate, 112.85 mg/dl of hexane, 99.17 mg/dl of chloroform and 109.67 mg/dl of acetone extract of *C. caudata*. Dhanasekaran et al., in their studies with ethanol crude extracts of medicinal plants, specifically with *C. argentea* showed 50% lethality of *Ae. aegypti* at its 134.4 ppm concentration [39]. When four solvent extracts like hexane, dichloromethane, ethyl acetate and diethyl ether of *Croton sparciflrorus* leaves were treated against three vectors *Ae. aegypti*, *An. stephensi* and *Cx. quinquefasciatus*, the LC_50_ values with the most effective ethyl acetate extract were recorded to be 34.02, 28.88 and 36.22 ppm respectively [40].

Plants belonging to the same family Magnoliaceae showed higher LC_50_ values (less potentiality) compared to the results obtained from *M. champaca*. As in case of *Magnolia salicifolia* fruits hexane and methanolic extracts caused 100% mortality at 250 ppm conc after 24 h of exposure of fourth instar larval stage of *Ae. aegypti* [18]. When *Ae. aegypti* larvae were exposed to the different parts of *Magnolia grandiflora*, worst toxic effects were observed at 49.4 and 48.9 ppm [19]. As per the study conducted by Luu-Dam et al., 100% mortality of 1st instar larvae of *Ae. aegypti* was recorded at 1000ppm concentration of the essential oil obtained from *Magnolia citrata* [17].

In the present study, 100% larval mortality was reported at 0.4% of the crude extract of *M. champaca* seeds within 72 h of treatment. From the phytochemical screening, the crude extract was found to contain tannin, terpenoids, saponin, flavonoids, alkaloids, coumarins, cardiac glycosides, and glycosides. The ethyl acetate extract of *M. champaca* seeds also showed promising larvicidal and pupicidal properties against *Ae. albopictus*, where 40 ppm ethyl acetate extract was enough to cause 100% larval mortality. The LC_50_ values obtained on ethyl acetate extract treatment were 0.98ppm, 4.50ppm, 5.41ppm and 7.40ppm for 1st, 2nd, 3rd and 4th instar larvae respectively, which are quite low compared to previous literature. Phytochemical analysis of the ethyl acetate extract showed the presence of tannin, terpenoids, saponin, flavonoids, alkaloids and coumarins. Regression analyses of both crude and ethyl acetate extractives depicted positive correlation between the extract concentration and mortality rates. Log-probit analyses at 95% confidence level represented that the LC_50_ and LC_90_ values gradually decreased with increasing concentration and exposure time. With respect to the pupal toxicity, an exposure to 200 ppm concentration of ethyl acetate seed extract for 48 h, resulted in complete mortality of 100%. The LC50 and LC90 values were found to be 62.38 ppm and 120.87 ppm respectively.

This is noteworthy, that under semi-field conditions ethyl acetate extractive of *M. champaca* exhibited impressive larvicidal potency against *Ae. albopictus* where 98% mortality was observed after 72 h of treatment at 500 ppm concentration. FT-IR analysis showed the presence of important functional groups such as aldehydes, aromatics, amine, amides, ketone, esters and anhydrides in the examined TLC fraction. The bioactive ingredient isolated from the TLC fractions by GC-MS analyses such as methyl 5,12-octadecadienoate and ethyl 9cis,11trans-octadecadienoate are specifically responsible for the larvicidal and the pupicidal activities against *Ae. albopictus.* The aforesaid compounds obtained after purification showed 100% larvicidal mortality at a very low concentration of 15ppm. Previously, saponin isolated from ethyl acetate extract of *Achyranthes aspera* caused mortality in *Ae. aegypti* larvae with LC_50_ value of 18.20 ppm [41]. Furthermore, the non-target organisms such as *Diplonychus* sp. and *Ischnura* sp. remained unaffected by the bio active ingredient isolated from *M. champaca*. During the pupal stage mosquito does not consume food. Thus, from considerable pupal mortality, it is assumed that the toxicity caused is due to absorption of active compounds by the body surface. Thus, the seeds of *M. champaca* demonstrated significant larvicidal and pupicidal effects on the dengue-transmitting mosquito species *Aedes albopictus*, while also displaying lower toxicity towards non-target organisms. It is noteworthy to state that the mosquitocidal properties of *M. champaca* against *Aedes albopictus* is totally a novel finding. In the future, there is still a need to explore the impact of the mentioned seed extract on different species of mosquitoes. It is necessary to study the effectiveness of the ethyl acetate extract from *M. champaca* seeds against *Ae. albopictus* under field conditions. The mechanism of action of the active bioingredients derived from the ethyl acetate extract of *M. champaca* seeds is yet to be investigated.

## Conclusion

In a nutshell, *M. champaca* seeds showed high degree of larvicidal and pupicidal activities against the dengue vector *Aedes albopictus* and also being less toxic to other non-target lifeforms indicating it to be eco-friendly. However, detailed field experimentation and evaluation of the most probable physiological mode of toxicity are to be revealed before commercial use.

### Limitations

All the experimentations had been done following the standard protocols of World Health Organization having standard sample size. Each of the experiments were triplicated with a set of control in all cases. Therefore, no such limitation is present in the data set.

## Data Availability

The databases used and analyzed during the current study is available from the corresponding author on reasonable request.
